# Peripartum Cardiomyopathy: an Update

**DOI:** 10.1007/s11897-018-0404-x

**Published:** 2018-07-26

**Authors:** Feriel Azibani, Karen Sliwa

**Affiliations:** 1 Hatter Institute for Cardiovascular Research in Africa, Faculty of Health Sciences, University of Cape Town, 4th floor Chris Barnard Building, Cape Town, South Africa; 2 Mary McKillop Institute, ACU, Melbourne, Australia

**Keywords:** Peripartum cardiomyopathy, Pregnancy, Biomarker, Ethnicity, Registry, Acute heart failure

## Abstract

**Purpose of Review:**

Peripartum cardiomyopathy (PPCM) is an idiopathic disorder defined as heart failure occurring in women during the last month of pregnancy and up to 5 months postpartum. In this review, we outline recent reports about the disease pathogenesis and management and highlight the use of diagnosis and prognosis biomarkers.

**Recent Findings:**

Novel data strengthen the implication of endothelial function in PPCM pathogenesis. The first international registry showed that patient presentations were similar globally, with heterogeneity in patient management and outcome.

**Summary:**

Despite large improvement in patient management and treatment, there is still a sub-group of women who die from PPCM or who will not recover their cardiac function. Remarkable advances in the comprehension of disease incidence, pathogenesis, and prognosis could be determined with multi-center and international registries.

**Clinical Trials:**

ClinicalTrials.gov Identifier: NCT02590601

## Introduction

It has been more than 150 years since the first description by Ritchie et al. [1] of idiopathic myocardial failure with onset in the puerperium [1] and in the postpartum period [2]. First described as a postpartum cardiomyopathy, the disease was recognized a few years later as a peripartum cardiomyopathy (PPCM) [3, 4]. PPCM is a maternal disorder which is well recognized in many countries where its incidence is high, such as Haiti [5], Nigeria [6], and South Africa [7].

Awareness about this disease has increased in the last few decades, with more systematic evaluation based on several national and international registries [8, 9]. However, despite remarkable advances in the comprehension of its pathogenesis [10] and improvement of patient management and therapy [11], PPCM is often recognized too late and it continues to be associated with high morbidity and mortality rates [12].

## Clinical Presentation, Definition, and Diagnosis

PPCM is defined as heart failure with unknown etiology, with onset of symptoms in a period comprising the last month of pregnancy and the first 5 months postpartum. A previous history of cardiac disease, or a family history of cardiomyopathy or sudden cardiac death, leads to the exclusion of a patient from diagnosis of PPCM [13]. Presenting symptoms in PPCM patients are highly variable but may include fatigue, dyspnea, orthopnea, peripheral edema, palpitations, chest pain, decreased exercise tolerance, and abdominal discomfort due to passive congestion of the liver.

Signs of both right and left heart failure including rales, jugular venous distension, gallop rhythm, ascites, and peripheral edema may be present. Blood pressure is usually normal or low due to low cardiac output. As pregnancy and the postpartum period are hypercoagulable states, patients may present with hemoptysis secondary to pulmonary embolism or neurologic symptoms due to an acute cerebrovascular event.

Most women with PPCM develop symptoms within the first few months following delivery, rather than during pregnancy [14–16]. As symptoms and signs of PPCM may mimic normal physiological findings of pregnancy and the postpartum period, the diagnosis of PPCM is often delayed.

Establishing the diagnosis of PPCM relies on a high index of suspicion, along with timing of symptom onset and imaging confirmation of LV systolic dysfunction.

As PPCM is considered to be a diagnosis of exclusion, a thorough evaluation is necessary in order to eliminate other potential cardiac and non-cardiac explanations for the patient’s clinical presentation.

The diagnostic algorithm suggested by the Heart Failure Association (HFA) Working Group on PPCM [11] requires clinical signs of heart failure and an echocardiographic left ventricular ejection fraction (LVEF) of ≤ 45%. ECG, magnetic resolution imaging (MRI), and laboratory measurement of NT-proBNP are not essential but are recommended for a better prediction of patient outcomes [16–19].

## Pathophysiology of PPCM

**Fig. 1:**
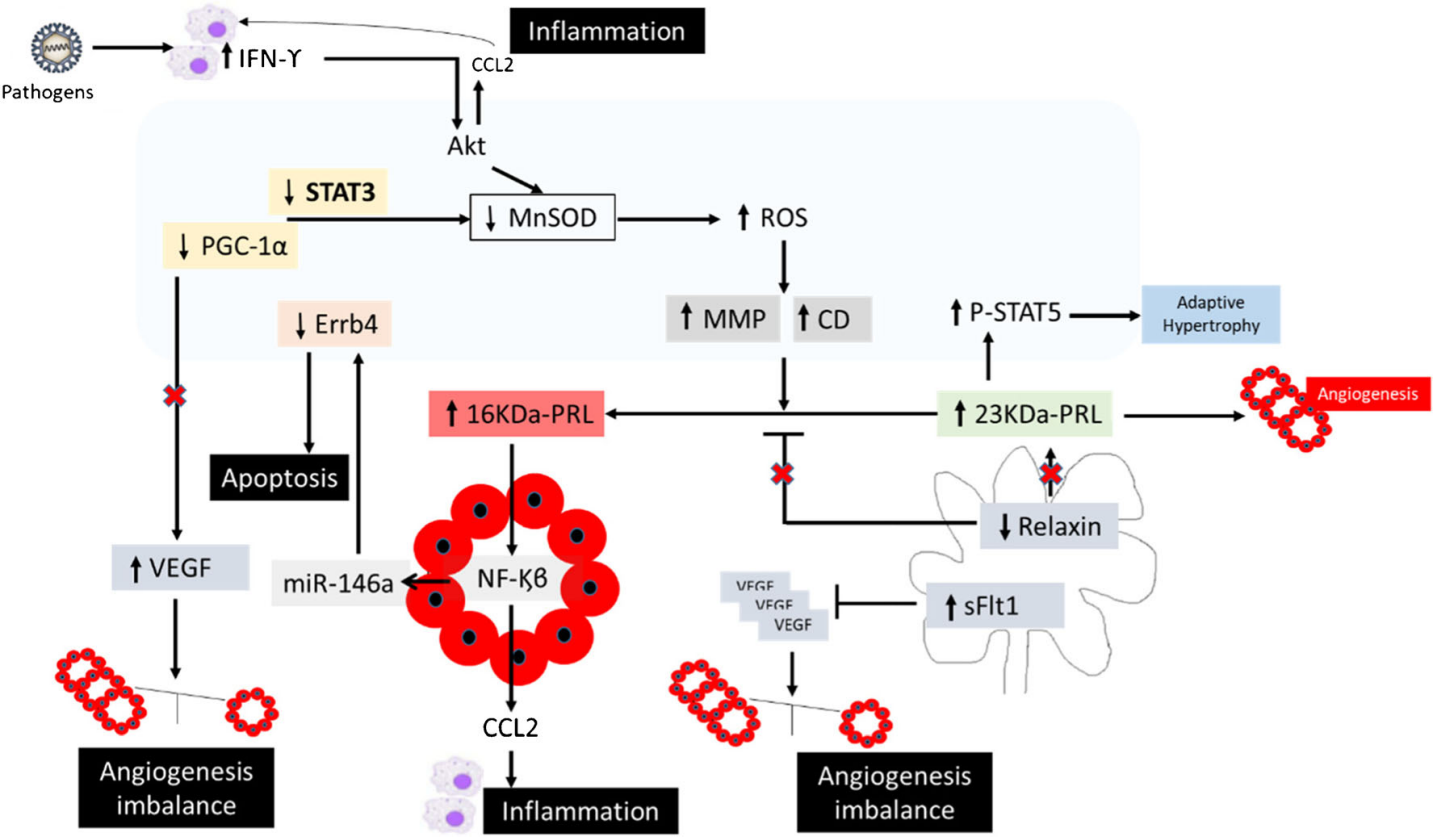
Pathophysiological mechanisms inducing peripartum cardiomyopathy: role of inflammation, apoptosis, and angiogenic imbalance. Decreased levels of STAT3 and/or PGC-1α increase ROS which will increase cathepsin D and MMP activity. Full-length prolactin (23KDa-PRL), highly expressed before delivery and postpartum, will be cleaved into 16KDa-PRL responsible of (1) increased expression of miR-146a which will inhibit endothelial cell proliferation and survival and induce cardiomyocyte apoptosis and (2) increased expression of CCL2 inducing cardiac inflammation. The angiogenic imbalance induced by 16KDa-PRL is augmented by increased sFlt1 and decreased VEGF and relaxin levels. Abbreviations: 16KDa-PRL, 16-kDa prolactin hormone; CCL2, chemokine ligand 2; CD, cathepsin D; Errb4, tyrosine kinase-type cell surface receptor HER4; IFN-ϒ, interferon-ϒ; MMP, matrix metalloproteinase; MnSOD, manganese-dependent superoxide dismutase; NFκB, nuclear factor-kappa B; PGC-1α, peroxisome proliferator-activated receptor gamma coactivator; PRL, prolactin hormone; ROS, reactive oxygen species; sFlt1, soluble fms-like tyrosine kinase-1; STAT, signal transducer and activator of transcription; VEGF, vascular endothelial growth factor

The precise mechanisms leading to cardiac dysfunction in human PPCM remain undefined. Increased oxidative stress, angiogenesis imbalance, and inflammatory reactions have been proposed as key features of the disease (Fig. 1).

Recently, the anti-angiogenic 16-kDa N-terminal prolactin fragment (16KDa-PRL), produced by cleavage of the full-length nursing hormone, prolactin (PRL), by cathepsin D, has been identified as a potential factor initiating and driving PPCM [20]. The hypothesis of its pathophysiological relevance was supported by the prior observation that treatment with the PRL blocker, bromocriptine, showed beneficial effects in some early clinical trials in PPCM patients [20–22]. The increase of 16KDa-PRL levels observed in PPCM patients has been attributed to the low activation of the signal transducer and activator of transcription 3 (STAT3) and the peroxisome proliferator-activated receptor gamma coactivator 1-alpha (PGC-1α). STAT 3 and PGC1-α have an essential role in proliferation and cell protection from death in the cardiac tissue [23, 24].

Cardiomyocyte specific deletion of STAT3 or PGC-α1 in rodents induces PPCM with a decrease of the anti-oxidant response in the heart, which is translated by decreased levels of anti-oxidant manganese-dependent superoxide dismutase (MnSOD) and increased reactive oxygen species (ROS) production. Increased oxidative stress subsequently activates cathepsin D, inducing the production of 16KDa-PRL.

In line with these experimental observations, increased levels of oxidized low-density lipoprotein (oxLDL), a marker of oxidative stress, and an increase of cathepsin D activity were associated with decreased STAT3 and PGC-1α expression in PPCM patients [20, 25, 26]. So far, the mechanism underlying downregulation of STAT3 and PGC-1α in PPCM patients has remained unknown.

The cleavage of prolactin into 16KDa-PRL may also be induced by matrix metalloproteinases (MMPs) (MMP-1, -2, -3, -8, -13) [27]. Interestingly, there is an increase of the circulating levels of MMP-2 in PPCM patients [25] and high expression levels of MMP-3 were found in the hearts of PPCM mouse models [20].

Endothelial dysfunction and angiogenic imbalance have also been investigated and appear to promote defect in oxygen supply to the cell leading to metabolic dysfunction in the heart, leading to cardiac contractility dysfunction in PPCM. Two distinct pathways mainly trigger the vascular impairment leading to heart failure. The first involves 16KDa-PRL—nuclear factor-kappa B (NFκB)—microRNA-146a pathway and the second is regulated by the balance between the anti-angiogenic factor the soluble fms-like tyrosine kinase-1 (sFlt1) and the pro-angiogenic factor vascular endothelial growth factor (VEGF). The 16KDa-PRL via the transcription factor NFκB upregulates endothelial expression of microRNA-146a. MicroRNA-146 inhibits proliferation and migration of endothelial cells. This microRNA can also be secreted in exosomes and can thus act on cardiomyocytes and induces metabolism dysfunction leading to their death [28].

PGC-1α downregulation results in insufficient upregulation of pro-angiogenic VEGF and, together with increased levels of anti-angiogenic soluble fms-like tyrosine kinase-1 (sFlt1) observed in the peripartum phase [26], aggravates the angiogenic imbalance. Interestingly, increased levels of circulating microRNA-146a, sFlt1, asymmetric dimethylarginine (ADMA), and apoptosis signaling molecules such as Fas/Apo1 (apoptosis signal receptor/apoptosis antigen 1) were observed in PPCM patients [16, 26, 28, 29].

Interestingly, Mebazaa et al. showed that there were increased levels of the pro-angiogenic factor placental growth factor (PLGF) in PPCM patients presenting early postpartum [30]. In a model of pressure overload, Seno et al. [31] demonstrated the efficacy of an anti-PlGF neutralizing antibody in the prevention of cardiac dysfunction. These results highlight the need for further exploration of the endothelial function in PPCM.

In addition to endothelial cell death and capillary rarefication, cardiomyocytes apoptosis via increased caspase 3 activation has also been reported in PPCM mouse models [32]. Inhibition of apoptosis in this model induced an improvement of cardiac function and an abolishment of mortality which support the causal link to pathogenesis of PPCM [33].

Current experimental and clinical advances in the understanding of PPCM pathophysiology highlight myocardial inflammation and autoimmune reactions as a possible underlying pathomechanism [16, 34]. Circulating levels of pro-inflammatory cytokines (interleukin-6, tumor necrosis factor-α, interferon-ϒ, and C-reactive protein) were found highly expressed and positively correlated with cardiac dysfunction [16, 35–37]. A shift towards humoral immunity is part of the physiological adaptation of the maternal body to pregnancy [38–40]. Interestingly, a recent study revealed a marked increase of innate immunity markers over humoral immunity in PPCM, suggesting disruption of immune homeostasis in PPCM [37].

In addition, reactive cardiac fibrosis has been demonstrated in PPCM animal models [20, 35]. Investigation of cardiac fibrosis in human PPCM using MRI showed that only a few women (10–15%) present with interstitial fibrosis [41–46]. These small cohort clinical studies should be confirmed in larger patient cohorts.

## Disease Management

Management of patients with PPCM is similar to that for patients with systolic heart failure due to other etiologies, but special consideration must be given to women who are either pregnant or breastfeeding. Patients should be managed by a multidisciplinary team including cardiologists, obstetricians, and neonatologists, among others.

As there are few controlled studies regarding the safety of heart failure medications used during pregnancy, treating patients diagnosed with PPCM during pregnancy presents a particular challenge.

### Initial Management

PPCM patients presenting with acute severe heart failure symptoms require prompt treatment in an intensive care unit. Noninvasive or mechanical ventilation may be necessary in some cases. Intravenous diuretics should be administered to patients with evidence of pulmonary congestion and volume overload. Temporary circulatory support with either intra-aortic balloon counter-pulsation or extracorporeal membrane oxygenation may be necessary in patients presenting with cardiogenic shock. Patients who clinically deteriorate, despite optimal medical treatment plus/minus temporary circulatory support, may benefit from implantation of a left ventricular assist device (LVAD), either as a bridge to recovery or transplantation [47]. As cardiac function can normalize within 6 months in a significant number of PPCM patients, the decision to refer the patient for cardiac transplantation should not be made too early. Outcomes after transplantation in PPCM patients are comparable to patients transplanted for other etiologies. A more recent study in a large collective of 42,406 patients, with 485 women transplanted due to PPCM, reports inferior graft survival, higher rejection rates, and higher mortality [48, 49].

### Chronic Heart Failure Management

During the antepartum period, heart failure therapy includes the use of diuretics to reduce preload and treat edema with a caution for dehydration-induced hypoperfusion [50] and vasodilators (hydralazine) to increase cardiac output and stroke volume and decrease vascular resistance [51], as well as beta-blockers. However, lower fetal birth weight [52] has been documented and should be monitored.

If the patient presents postpartum, patients should be managed according to standard heart failure guidelines [53]. Neurohormonal blockade with angiotensin-converting enzyme inhibition, angiotensin receptor blockers, and mineralocorticoid receptor blockers are considered as first-line heart failure medication [53].

### Use of Prolactin Inhibitor Bromocriptine

Numerous recent case reports have indicated that the addition of bromocriptine, a drug inhibiting prolactin, to standard heart failure therapy may be beneficial in patients with acute onset PPCM. A proof-of-concept pilot study of PPCM patients with severely reduced LVEF diagnosed within 1 month of delivery showed better recovery of left ventricular function in patients treated with bromocriptine, compared with patients receiving placebo [22]. A larger, randomized multi-center study from Germany confirmed the previously reported beneficial effects of bromocriptine. [21, 22]. All patients receiving bromocriptine should receive standard heart failure therapy with at least prophylactic anticoagulation. This treatment regime has recently been termed the BOARD (Bromocriptine, Oral heart failure therapies, Anticoagulation, vasoRelaxing agents, and Diuretics) regime [54]. However, as with all other medication, there needs to be a risk/benefit assessment. In patients with milder left ventricular dysfunction, one might feel that continuation of breastfeeding may provide better long-term benefits for the infant, versus accepting the possibility of remaining with some degree of left ventricular function dysfunction. Overall, the evidence of using bromocriptine in patients diagnosed with PPCM is based on a limited number of smaller studies. In Canada, a placebo-controlled study is under way, albeit again with limited patient numbers (ClinicalTrials.gov Identifier: NCT02590601). The large prospective international EORP registry on PPCM patients with information on treatment and outcome which included over 750 patients has just been completed recruitment [55]. From this large international PPCM collective, we expect more data on the efficacy of bromocriptine treatment.

### Anticoagulation

Women presenting with PPCM are vulnerable to thrombotic and thromboembolic complications due to *pregnancy-induced hypercoagulability which begins during pregnancy, is heightened during labor and delivery, and persists up to* several months postpartum. These patients not only have an increased risk of deep vein thrombosis and pulmonary embolism but also have an increased risk for developing intracardiac thrombus, even if their LVEF is only moderately decreased. Anticoagulation is not recommended for all PPCM patients but should be considered in patients with an LVEF < 35%.

### Antiarrhythmic Device Therapy

Beta-blockers and non-vasoselective calcium-channel blockers may safely be used for rate control of tachyarrhythmias. There are no guidelines for implantation of an implantable cardiac defibrillator (ICD) specifically for PPCM patients. Sudden cardiac death has been reported in PPCM patients with decreased LVEF in both the acute and chronic stages of this disease, as well as in those whose LVEF has completely normalized, indicating that the risk of sudden cardiac death may persist well into recovery in this patient population. Patients with sustained ventricular arrhythmias or history of sudden cardiac death in the acute setting may be candidates for ICD implantation, but this decision must be carefully weighed against the evidence that LV function improves within 6 months in the majority of PPCM patients. It would not be unreasonable to consider ICD implantation in patients with persistent NYHA class III or IV symptoms despite optimal medical therapy for 6 months and whose LVEF remains < 30%. A suitable alternative is wearable cardioverter-defibrillator devices for patients with LVEF ≤ 35% to prevent sudden cardiac death [56, 57].

Cardiac resynchronization therapy may be considered in PPCM patients with chronic heart failure if, despite optimal medical therapy, they have persistent NYHA class III or IV symptoms, LVEF < 35%, and QRS duration ≥ 120 ms.

## Circulating Biomarkers of PPCM

Diagnosis of PPCM based on exclusion is a difficult task. PPCM patients present with symptoms of heart failure and signs of systolic dysfunction (i.e., palpitations, dizziness, and chest pain), which delays PPCM diagnosis [13, 58–60].

The quality of a biomarker relies on its sensitivity and specificity to detect the disease. Despite its sensitivity to diagnose PPCM [15, 25, 47, 61–63] and cost-effectiveness, natriuretic peptides (brain natriuretic peptide “BNP” and N-terminal-proBNP “NT-proBNP”) are, unfortunately, non-specific markers for a variety of other cardiovascular pathologies, including myocardial ischemia, preeclampsia, pulmonary emboli, and, most notably, heart failure [15, 47, 64, 65]. However, when the disease presentation is mild, dosage of natriuretic peptides can be useful, in association with echocardiography, hence hastening the diagnosis of heart failure [66]. Currently, only these markers are used in routine clinical diagnosis of PPCM.

The lack of specificity seems to be resolved with the use of microRNA-146 whose expression is higher in PPCM patients, when compared to dilated cardiomyopathy patients [15, 28]. In addition, circulating endothelial and monocyte microparticles were found specifically in PPCM women, when compared to healthy pregnant and postpartum women and to patients with other heart diseases [67].

As per the disease pathology, prolactin and cathepsin D levels were increased in PPCM patients compared to healthy matched controls [15, 25]. Interestingly, higher levels of 16KDa-PRL and increased cathepsin D activity were observed in a small cohort of PPCM patients during their acute phase [20], but this should be validated in a larger cohort.

Soluble Flt1 is increased in PPCM women with higher NYHA functional class at presentation [68]. However, sFlt1 is secreted by endothelial cells and the placenta in late pregnancy [69] and its levels usually drop rapidly postpartum [70, 71]. Similarly, relaxin-2, another pregnancy-related biomarker, is decreased in PPCM patients [72]. It is thus challenging to establish a clear threshold value because of its pregnancy-specific variability.

Another biomarker of endothelial function and oxidative stress, oxidized LDL (oxLDL), and mid-regional pro-adrenomedullin (MR-proADM) were found to be increased in PPCM women [25, 30].

Cardiac remodeling biomarkers were shown to be useful in the diagnosis of PPCM. Decreased levels of transforming growth factor-β [25, 73], together with increased levels of MMP2 [74], TNF-α [25, 75, 16, 29], interleukin-6 [29], interleukin-4 [37], interferon-ϒ [25, 37], soluble ST2 [30], and Fas/Apo1 [16, 25, 29] are associated with PPCM.

The maternal outcome in PPCM is a major concern as a high percentage of women will not recover their cardiac function [5, 9, 14, 16, 19], leading to death in 1.4 to 28% [9, 16, 19, 29, 76–82]. Various circulating biomarkers were tested for their potential to predict the 6-month postpartum maternal outcome (Table 1).

**Table 1:**
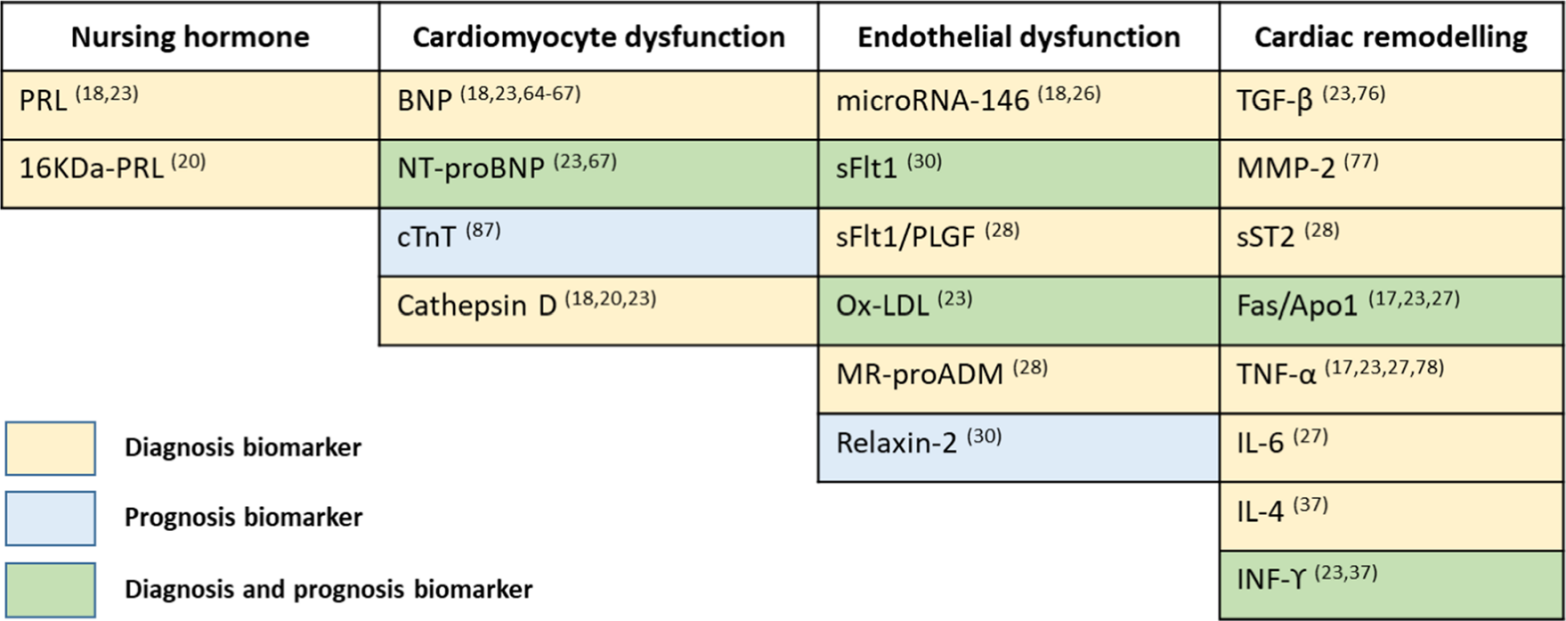
Overview of diagnosis and prognosis biomarkers in peripartum cardiomyopathy

*Abbreviations*: *16KDa-PRL*, 16-kDa prolactin hormone; *Apo1*, apoptosis antigen 1; *BNP*, brain natriuretic peptide; *cTnT*, cardiac troponin I; *IFN-ϒ*, interferon-ϒ; *IL*, interleukin; *MMP-2*, matrix metalloproteinase 2; *MR-proADM*, mid-regional pro-adrenomedullin; *NT-proBNP*, N-terminal pro-brain natriuretic peptide; *OxLDL*, oxidized low-density lipoprotein; *PLGF*, placental growth factor; *PRL*, prolactin hormone; *sFlt1*, soluble fms-like tyrosine kinase-1; *sST2*, soluble ST2; *TGF-β*, transforming growth factor-β; *TNF-α*, tumor necrosis factor-α

Baseline NT-proBNP levels in PPCM are higher in patients who fail to improve within the initial 6 months postpartum [25, 63]. Baseline total cholesterol is directly correlated with cardiac function at 6 months [16, 83].

Serum Fas/Apo1, measured at patient presentation, failed to predict improvement of cardiac function in PPCM patients after 6 months, but was significantly successful in predicting which patients would die and which would survive [16, 25, 29]. Additionally, elevated serum cTnT was able to foresee normalization of LVEF with a strong specificity and moderate sensitivity [84]. Higher sFLT1 levels were associated with more severe functional limitations and major adverse clinical events [68].

In addition, median interferon-gamma (IFN-ϒ) is significantly decreased 6 months postpartum in improvers, but not in non-improvers, and its changes correlated negatively with corresponding changes in EF. Hence, like oxLDL, IFN-ϒ can be used as a biomarker to monitor disease progression [25].

Higher relaxin-2 levels were associated with more rapid myocardial recovery (higher LVEF at 2 months) [68]. However, Nonhoff et al. did not see any correlation between relaxin-2 levels and faster improvement of PPCM patients [85].

## Subsequent Pregnancies

PPCM has been described in primiparous and multiparous women. Several studies highlighted the higher incidence of PPCM in women with high parity and gravidity [5, 29, 55]. In addition, there is clear evidence of increased risk in subsequent pregnancy in PPCM patients. Thus, increased morbidity and mortality is observed during subsequent pregnancies in PPCM patients, especially in women with persistent left ventricular dysfunction after the first pregnancy [86–89]. A recent review highlighted the urgent need of early contraception in PPCM women [90].

## Learnings from National and Worldwide Registries

PPCM is a heterogeneous disorder, very often described in small cohorts which lead to limited results interpretation and conclusions. In 2014, the European Society of Cardiology (ESC) Working Group on Peripartum Cardiomyopathy initiated the first worldwide registry on PPCM [8], which included 61 countries, of which 43 are active, and more than 140 centers participated in the registry. The EURObservational PPCM Registry aims to describe PPCM patient presentation, comorbidities, diagnostics, and management. Interestingly, analysis of the first 500 PPCM patients recruited to the registry showed exactly the same patient presentation and baseline characteristics in both ESC and non-ESC-affiliated countries, despite differences in ethnicity and socio-demographic parameters [55]. However, patient outcomes were different, with a higher proportion of persistent heart failure 1 year postpartum in non-ESC-affiliated countries. The Pregnancy-Associated Cardiomyopathy (IPAC) Working Group was the first multi-center PPCM network—initiated in 2009 as a National Heart, Lung, and Blood Institute funded multi-center—to investigate PPCM patient characteristics, treatment, and clinical predictors of outcome in North America [9]. Reports from this working group first highlighted a moderate recovery rate with 13% of affected women having major events (death, cardiac transplantation, and implantation of a LVAD) or persistent cardiomyopathy, with severe left ventricular dysfunction and greater remodeling at baseline being associated with less recovery [9]. A second study reported the presence of right ventricular (RV) dysfunction in one third of PPCM patients which was an independent predictor of subsequent lack of recovery of LV function and clinical outcomes. LV systolic dysfunction severity did not predict the severity of RV systolic dysfunction and thus poor outcome [91]. Genetic and MRI analyses of the IPAC cohort showed that polymorphism in GNB3 gene was highly associated with lower LVEF at follow-up, particularly evident in black women [92], and only a small minority of patients showed focal myocardial damage contributing to persistent myocardial dysfunction [41].

Other population-based studies and national registries contributed to characterize patient presentation and outcome. The German PPCM registry described patients mostly presenting at delivery or within the first postpartum month, with 15% of patients not improving their cardiac function [15]. The study of risk factors showed that smoking, multiple parities, and twin pregnancies may increase the risk for PPCM. Interestingly, German patients suffering from PPCM with concomitant hypertension had a good recovery rate (97% of hypertensive patients). In the Japanese cohort, hypertension-induced protection from bad outcome was milder, with the same death and hospitalization rate when compared to the non-hypertensive PPCM women [93]. However, the investigators observed a shorter duration of hospitalization in PPCM patients with hypertension compared to the non-hypertensive PPCM women.

Population-based studies of PPCM demonstrated that the incidence of PPCM was 1 in 1741 deliveries in South Korea [94] and 1 in 20,000 deliveries in Japan [93]. The outcome was also different between the two populations, with a high in-hospital mortality in South Korea [94] and 4% mortality in Japan [93].

Very recently, the A RegisTry of pEripartuM cardIomyopathy in Turkish patientS (ARTEMIS) program was designed to be a PPCM registry for Turkey, planned under the umbrella of the worldwide PPCM registry [95].

## Conclusion

PPCM is a rare but serious disorder associated with high morbidity and mortality that occurs worldwide, although with different prevalence and incidence rates. Despite a large improvement in patient management and outcome, efforts are still needed to better understand why and how the disease can affect specific women, and which parameters affect the improvement of cardiac function in the sub-group of women with bad outcome. Special attention should be paid to evaluating the role of ethnicity in the pathogenesis of the disease. More knowledge will be derived from national multi-center and worldwide registries in the future.
